# The role of donor hypertension and angiotensin II in the occurrence of early pancreas allograft thrombosis

**DOI:** 10.3389/fimmu.2024.1359381

**Published:** 2024-05-17

**Authors:** Christophe Masset, Julien Branchereau, Fanny Buron, Georges Karam, Maud Rabeyrin, Karine Renaudin, Florent Le Borgne, Lionel Badet, Xavier Matillon, Christophe Legendre, Denis Glotz, Corinne Antoine, Magali Giral, Jacques Dantal, Diego Cantarovich, DIVAT Consortium, Lionel Badet

**Affiliations:** ^1^ Institut de Transplantation-Urologie-Néphrologie (ITUN), Nantes University Hospital, Nantes, France; ^2^ Nantes Université, INSERM, Center for Research in Transplantation and Translational Immunology, UMR 1064, Nantes, France; ^3^ Groupement Hospitalier Edouard Herriot Service d’urologie chirurgie de la transplantation, Lyon, France; ^4^ Groupement Hospitalier Edouard Herriot, Service d’anatomie et pathologie, Lyon, France; ^5^ Service d’anatomie et pathologie, CHU de Nantes, Nantes, France; ^6^ INSERM UMR 1246 - SPHERE, Nantes University, Nantes, France; ^7^ Department of Nephrology and Kidney Transplantation, Necker Hospital, Assistance Publique-Hôpitaux de Paris, Paris, France; ^8^ Institut de Recherche Saint Louis, INSERM U976, Paris, France

**Keywords:** body mass index (BMI), pre-procurement pancreas suitability score, pancreas transplantation, allograft thrombosis, high blood pressure, immunothrombosis

## Abstract

**Background:**

About 10–20% of pancreas allografts are still lost in the early postoperative period despite the identification of numerous detrimental risk factors that correlate with graft thrombosis.

**Methods:**

We conducted a multicenter study including 899 pancreas transplant recipients between 2000 and 2018. Early pancreas failure due to complete thrombosis, long-term pancreas, kidney and patient survivals were analyzed and adjusted to donor, recipient and perioperative variables using a multivariate cause-specific Cox model stratified to transplant centers.

**Results:**

Pancreas from donors with history of hypertension (6.7%), as well as with high body mass index (BMI), were independently associated with an increased risk of pancreas failure within the first 30 post-operative days (respectively, HR= 2.57, 95% CI from 1.35 to 4.89 and HR= 1.11, 95% CI from 1.04 to 1.19). Interaction term between hypertension and BMI was negative. Donor hypertension also impacted long-term pancreas survival (HR= 1.88, 95% CI from 1.13 to 3.12). However, when pancreas survival was calculated after the postoperative day 30, donor hypertension was no longer a significant risk factor (HR= 1.22, 95% CI from 0.47 to 3.15). A lower pancreas survival was observed in patients receiving a pancreas from a hypertensive donor without RAAS (Renin Angiotensin Aldosterone System) blockers compared to others (50% vs 14%, p < 0.001). Pancreas survival was similar among non-hypertensive donors and hypertensive ones under RAAS blockers.

**Conclusion:**

Donor hypertension was a significant and independent risk factor of pancreas failure. The well-known pathogenic role of renin-angiotensin-aldosterone system seems to be involved in the genesis of this immediate graft failure.

## Introduction

Pancreas transplantation can provide higher patient survival and better quality of life in selected patients with diabetes (1, 2). However, about 10% to 20% of patients will prematurely lose their pancreas allograft due to acute thrombosis (3) (also historically defined as “technical failure”). This major postoperative complication which induces high morbidity and mortality, is rarely observed for other solid organ transplants (4, 5). So far, one of the main research objectives in the field of pancreas transplantation is the prevention of early allograft failure. Many studies performed in the early 2000’s have identified potential risk factors associated with early pancreas failure. Among them, the main one was a cold ischemia time over 10–12 hours (6). Elevated body mass index (BMI) in the donor was also recurrently associated with acute pancreas thrombosis (7), notably when above 30 kg/m^2^. Advanced donor age is amongst the top three major risk factors (8, 9). Vascular cause of death (10), hemodynamic instability during the organ procurement (11) and even surgeon’s experience (12) complete the list (13). Whilst mechanisms favoring thrombosis may be different according to each risk variable identified, in the end all of them induce an inflammation of the gland and consequently partial or total thrombosis may occur. Therefore, a strict selection of donors and recipients is highly recommended before performing transplantation to reduce the occurrence of thrombosis. Two pretransplant scores, established in this regard, are available: the Pre-procurement Pancreas Suitability Score (P-PASS) (13) and the Pancreas Donor Risk Index (PDRI) (14). Unfortunately, despite major efforts to select the best pancreas donor, the occurrence of early allograft failure has essentially remained unchanged, suggesting we are still faced with mechanisms that need to be investigated (15, 16). The mitigating results of both P-PASS and PDRI scores on prospective cohorts have reinforced this hypothesis (17–19).

The primary objective of our study was to search for potential new risk factors in the donor and during the immediate postoperative period that could be associated with early pancreas failure, i.e. pancreas removal due to complete thrombosis. For this purpose, a large cohort of patients was evaluated among four main pancreas transplant centers in France.

## Methods

### Studied population

All patients receiving a pancreas transplant between January 1^st^ 2000, and December 31^st^ 2018 in four major French university hospital centers were included (Nantes, Lyon, Paris Necker and Paris Saint Louis). We categorized pancreas transplants as simultaneous pancreas-kidney (SPK), pancreas after kidney (PAK) and pancreas transplant alone (PTA). All data were extracted from the French multicenter observational and prospective DIVAT cohort of transplanted patients (www.divat.fr).

### Organ allocation and transplantation procedure

All pancreas transplant were harvested from brain dead deceased donors less than 50 years old (45 years old between 2000 and 2013) and with a BMI < 30 mg/kg. Diabetic and/or alcoholic patients are excluded from the pancreas donation program. In case of poor macroscopic appearance of the pancreas allograft (ischemia, edematous or fatty presentation) the organ was not transplanted. A national priority is given for patients waiting for a SPK. All allografts were allocated nationally by the French Agency of Biomedicine.

Management of pancreas transplantation was similar for all types of categories (SPK, PAK and PTA) and remained broadly similar during the study period in all centers. Donor duodenal anastomosis was performed for exocrine diversion. In most cases, induction therapy consisted of antithymocyte globulin for 5 days, associated with two pulses of 500mg of methylprednisolone. Maintenance immunosuppressive therapy consisted of the association of calcineurin inhibitor (mainly tacrolimus) and mycophenolate mofetil or mycophenolic acid, following the standard guidelines of dosage. Oral prednisone was given according to center’s policy. The anticoagulation protocol consisted of per-operative administration of intravenous aspirin (250mg) and heparin (25 UI/kg) at the time of clamping; followed by preventive anticoagulation using low molecular weight heparin within the first days after surgery (mostly 10 days). In case of partial thrombosis, a curative anticoagulation was administered (heparin, later converted to vitamin K antagonists). In the absence of allograft thrombosis, detected on a systematic CT-scan at Day 10, preventive heparin was replaced by 100mg of aspirin which was continued long-term.

### Available data

Complete available data is presented in **Table 1**. The donor’s considered biological values, such as lipasemia or estimated glomerular function (eGFR), were those obtained the closest to the surgery. Hypertension in the donor was considered by collecting medical records and interrogation of the referring physician and/or donor’s family during organ allocation. Recipient screening for biological thrombophilia was performed after occurrence of pancreas thrombosis – although not systematically; and/or in patients with pretransplant risk factors (history of venous thrombosis, spontaneous increase in APTT). The follow-up and the collection of data stopped upon transplant failure (pancreas failure for PTA or return to chronic dialysis and pancreas failure for SPK) or death.

**Table 1:** Descriptive table of studied patients comparing those with an early pancreas failure and those with a functional allograft at D30 post-transplantation (p-values are obtained using Chi-square test or Fisher exact test for categorical variables and using Student’s t-test or Mann-Whitney U for continuous variables).

	Whole cohort (n=899)			Early Failure (n=122)			Success (n= 777)			p-value
	NA	n	%	NA	n	%	NA	n	%	
Type of graft	0			0			0			
* SPK*		784	87.2		105	86.0		679	87.4	0.6845
* PAK*		55	6.1		9	7.4		46	5.9	0.5325
* PTA*		60	6.7		8	6.6		52	6.7	0.9557
Male recipient	0	510	56.7	0	71	58.2	0	439	56.5	0.7250
Re-transplantation	0	59	6.6	0	11	9.0	0	48	6.2	0.2391
History of hypertension	0	762	84.7	0	105	86.0	0	657	84.5	0.6662
History of vascular disease	0	211	23.5	0	31	25.4	0	180	23.1	0.5867
History of cardiac disease	0	196	21.8	0	24	19.7	0	172	22.1	0.8658
Pancreas conservation fluid preservation fluid	44			9			35			
* Celsior*		237	27.8		32	28.3		205	27.6	0.8786
* IGL*		320	37.4		46	40.7		274	36.9	0.4391
* Other*		298	34.8		35	31.0		263	35.4	0.3528
Male donor	2	586	65.3	0	81	66.3	2	505	65.2	0.7904
Exocrine derivation	326			50			276			
* Enteric with Roux*		269	47.0		29	40.3		240	47.9	0.2253
* Enteric without Roux*		226	39.4		30	41.7		196	39.1	0.6795
* Other*		78	13.6		13	18.0		65	13.0	0.2397
Vascular cause of donor death	3	341	38.0	0	47	38.5	3	294	38.0	0.9091
Donor hypertension history	51	48	5.7	6	14	12.1	45	34	4.6	0.0013
History of donor dyslipidemia	237	13	1.9	29	1	1.1	208	12	2.1	0.5054
History of cardiac arrest before sampling	8	150	16.8	1	16	13.2	7	134	17.4	0.2534
Vasopressive drug	40	753	87.6	7	100	87.0	33	653	87.7	0.8053
HLA-A-B-DR incompatibilities > 4	7	430	48.2	4	58	49.1	3	372	48.1	0.8252
Depleting induction	7	848	95.1	2	113	94.2	5	735	95.2	0.6243
	NA	m	SD	NA	m	SD	NA	m	SD	p-value
Recipient age (years)	1	39.7	7.9	1	40.5	8.6	0	39.6	7.8	0.2414
Recipient BMI (kg/m²)	10	22.8	3.1	3	23.2	3.7	7	22.8	3.1	0.3726
Pancreas cold ischemia time (min)	83	674	163	17	693	182	66	671	159	0.1913
Kidney ischemia time in SPK (min)	78	790	179	18	809	189	60	787	178	0.2464
Duration in post-op ICU (days)	280	2.5	3.7	34	3.9	6.9	246	2.2	2.7	0.0010
Donor age (years)	4	32.5	10.4	1	33.7	10.1	3	32.3	10.4	0.1725
Donor BMI (kg/m²)	34	22.9	2.9	5	23.7	3.3	29	22.7	2.8	0.0005
Donor eGFR (MDRD, ml/min/m^2^)	27	111	44	4	117	38	23	110	45	0.1477
Donor lipasemia (IU/L)	286	63	393	28	56	101	258	65	425	0.5093

BMI, body mass index; CMV, cytomegalovirus; DSA, donor-specific antibodies; eGFR, estimated glomerular filtration rate; HLA, human leucocyte antigens; ICU, intensive care unit, NA, not available (missing); PAK, pancreas after kidney; PTA, pancreas transplant alone; SD, standard deviation; SPK, simultaneous pancreas-kidney.

### Studied endpoints

The primary endpoint was pancreas early allograft failure due to thrombosis, defined as pancreas allograft removal or permanent exogeneous insulin dependence state on postoperative day 30 and thereafter. In addition, secondary endpoints included the long-term pancreas survival (20), kidney survival (in case of SPK) and patient survival.

### Statistical analyses

The characteristics at transplantation were described using frequency and proportion for categorical variables and mean and standard deviation for continuous variables. The median time of follow-up was estimated by using the reverse Kaplan-Meier (21). The cumulative incidence curves of the studied outcomes were obtained by the Aalen-Johansen estimator to account for competing events (22). For graft survival criteria, deaths were considered competing events in the Aalen-Johansen estimator and were right-censored in the cause-specific Cox models. To estimate the relationship between the different potential risk factors and the studied outcomes we used a multivariate cause-specific Cox model stratified on centers (23). Competing events were right-censored (24). The hazard proportionality assumption was graphically verified. If this assumption did not hold, two different periods were considered. For continuous risk factors, the log-linearity assumption was checked in univariate analysis if the Bayesian Information Criterion was not reduced using natural spline transformation compared to the inclusion of the variable in its natural scale. In case of violation, variables were categorized. Significant variables in univariate analysis (P< 0.20) were further analyzed in a multivariate model to determine those acting independently (descending procedure, P< 0.05). Based on theoretical considerations, we entered the following variables in the multivariable models: donor age, donor body mass index (BMI), vascular cause of death and pancreas cold ischemia time. As hypertension may correlate with BMI, the interaction term was tested. Patients with missing data of the retained covariates in the multivariable models were excluded. For the study on kidney graft failure, we excluded patients who received a pancreas transplant alone. We described the characteristics of the studied patients and those of the excluded patients.

We used R version 3.6.1 (25) and ‘base’, ‘dplyr’, ‘survival’, ‘etm’, ‘plotrix’, ‘splines’, ‘lattice’, ‘prodlim’, ‘ReporteRs’, ‘ggplot2’ and ‘tidyverse’ packages for all data analyses.

### Ethics statement

Data were extracted from the French DIVAT cohort (www.divat.fr, approved by the independent ethic committee, CNIL n°914184) consisting of pancreas transplant recipients monitored in Nantes, Lyon, and Paris Necker and Paris St Louis. The quality of the DIVAT data bank is validated by an annual cross-center audit. All participants gave informed consent, and data were de-identified to respect confidentiality.

## Results

### Recipient demographic characteristics

During the study period, 915 pancreas transplantations were performed, among whom 16 were excluded because of missing data on pancreas survival. We thus evaluated 899 recipients of pancreas transplantation categorized as Simultaneous Pancreas-Kidney (SPK), n= 784 (87.2%), Pancreas after Kidney (PAK), n= 54 (6.0%) and Pancreas Transplant Alone (PTA), n= 58 (6.5%). Characteristics of all patients are described in **Table 1**. The median time of follow-up of all 899 recipients was 4.91 years (range from 0.1 to 20.0 years). During the first 30 postoperative days, we observed 122 (13.6%) pancreas failures. In 19 patients the cause of failure was missing; 90 were due to complete thrombosis (88.2%), 9 to hemorrhage (8.8%) and 3 to pancreatitis (3.0%). Among pancreases explanted in the first month, histological patterns of rejection were suspected in 2 patients. Screening for biological factors at risk of thrombosis was performed in 41 of thrombosed cases; 3 of them were positive (7.3%). Six patients died with a functional allograft during the first 30 days. During the complete follow-up, there were 85 deaths (9.4%) with at least one functioning graft, 219 pancreas failures (24.3%) and 107 kidney graft failures (11.9%).

### Risk factors for early pancreas failure

**Figure 1:**
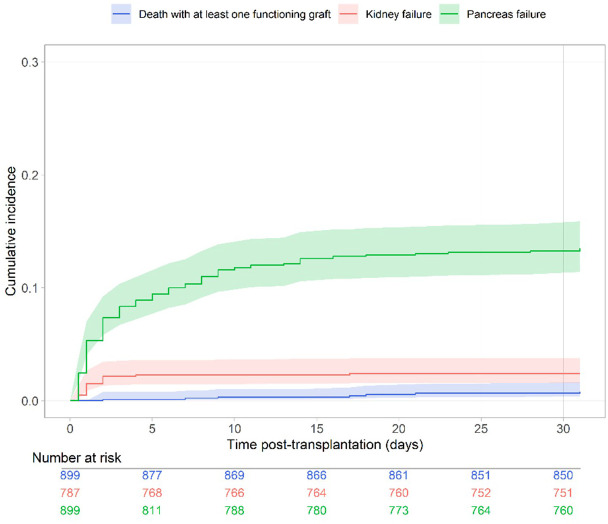
Cumulative incidence of pancreas failure, kidney failure and death during the first 30 days after transplantation (Aalen-Johansen estimator).

The cumulative incidence of pancreas failure within the first month after surgery is presented in **Figure 1**. The cumulative incidence rate at 5, 10 and 30 days was 8.9% (95% CI from 7.2% to 11.0%), 11.6% (95% CI from 9.7% to 13.9%), and 13.5% (95% CI from 11.4% to 15.9%), respectively. In the multivariate analyses, 113 observations were deleted due to missing data of covariates, their characteristics are described in the **Supplementary Table S1**. **Supplementary Table S2** presents the unadjusted association between the potential risk factors and early pancreas failure. The final multivariate Cox model stratified to centers is presented in **Table 2**. We observed that the risk of early pancreas failure with the current selection of donors was not associated with cold ischemia time, donor’s age nor vascular cause of death. However, two factors remained significantly associated with an increased risk of early pancreas failure: donor hypertension (HR= 2.57, 95% CI from 1.35 to 4.89) and donor’s BMI (for an increase of 3 kg/m² the HR was equal to 1.11 95% CI from 1.04 to 1.19). Because hypertension may correlate with BMI, we assessed the interaction between hypertension and BMI in the donor, which was negative.

**Table 2:** Multivariable cause-specific Cox model associated with the risk of pancreas graft failure within the first 30 days post-transplantation (n= 786, 113 observations removed because of missing data).

	HR	95% CI	p-value
Pancreas cold ischemia time (hours)	1.05	[0.98; 1.14]	0.1773
Donor age (years)	1.01	[0.98; 1.03]	0.6149
Donor BMI (kg/m²)	1.11	[1.04; 1.19]	0.0020
Donor history of hypertension	2.57	[1.35; 4.89]	0.0039
Vascular cause of donor death	1.16	[0.53; 1.41]	0.5616

BMI, body mass index; CI, confidence interval; HR, hazard ratio. The model was stratified on the center.

### Risk factors for long-term pancreas failure

**Figure 2:**
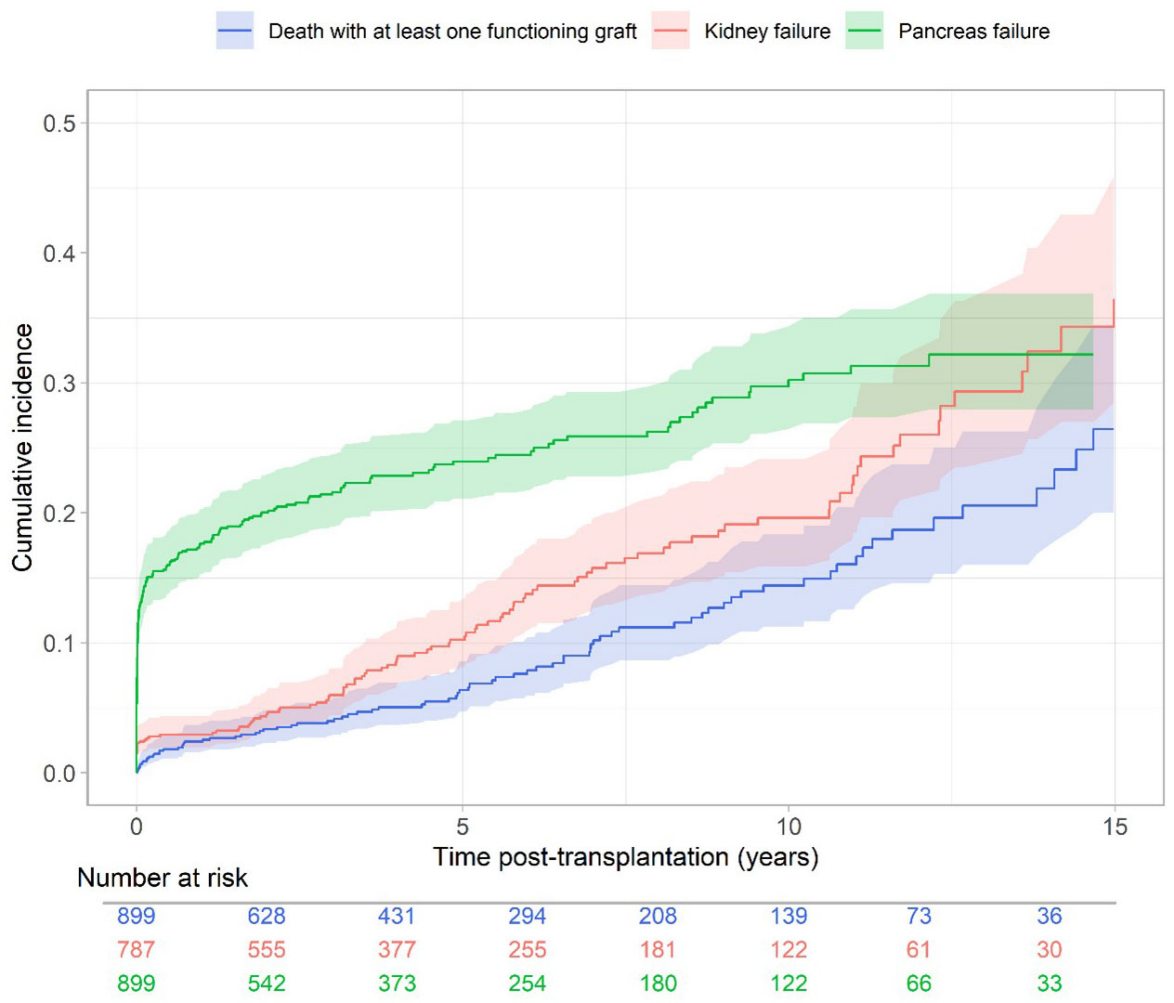
Cumulative incidence of long-term pancreas failure, renal graft failure and death (Aalen-Johansen estimator).

The cumulative incidence of long-term pancreas failure is presented in **Figure 2**. Cumulative incidence rate at 5 and 10 years was 23.9% (95% CI from 21.1% to 27.1%) and 30.2% (95% CI from 26.5% to 34.3%), respectively. The unadjusted and confounder-adjusted associations between the covariates and long-term pancreas failure is presented in **Supplementary Tables S3** and **S4**. We observed a significant increase in the long-term risk of pancreas failure in recipients of an allograft from a donor with hypertension (HR= 1.88, 95% CI from 1.13 to 3.12) or a donor with high BMI (for an increase of 3 kg/m² in the donor BMI the HR was equal to 1.21, 95% CI from 1.04 to 1.41). We also observed a significant increase in the risk of pancreas failure in recipients of a PTA compared to SPK (HR= 2.52, 95% CI from 1.59 to 3.98).

Considering only patients with a functional pancreas allograft after postoperative day 30 (i.e., insulin independence), the only risk factor for long-term pancreas failure was PTA (HR= 5.03, 95% CI from 2.76 to 9.16), **Supplementary Tables S5** and **S6**. Donor hypertension and obesity were no longer associated with pancreas failure from postoperative day 31 and thereafter (respectively, HR= 1.22, 95% CI from 0.47 to 3.15 and HR= 0.99, 95% CI from 0.92 to 1.07), meaning that those parameters only influenced the risk of immediate thrombosis.

### Risk factors for kidney allograft failure and patient death

The cumulative incidence of kidney failure among the 787 recipients of a SPK transplant is presented in **Figure 2**. **Supplementary Tables S7**, **S8** show the unadjusted and confounder-adjusted associations between the covariates and kidney failure and patient death respectively. We observed a significant increase in the risk of kidney failure for an increase of 3 kg/m² in donor BMI (the HR was equal to 1.29, 95% CI from 1.04 to 1.59, p= 0.0180), but not for a donor with hypertension (HR= 1.58, 95% CI from 0.80 to 3.10 p = 0.1844). In the multivariate analyses, 50 observations were deleted due to missing data of covariates (**Supplementary Table S9**).

The cumulative incidence of patient death is presented in **Figure 2**. **Supplementary Tables S10**, **S11** present the unadjusted and confounder-adjusted associations between the covariates and death. We observed a two-fold increase in the risk of patient death in recipients with history of vascular disease (HR= 2.01, 95% CI from 1.24 to 3.26) and an increase in the risk of death for older recipients (for an increase of 5 years the HR was equal to 1.29, 95% CI from 1.11 to 1.50). In the multivariate analyses, 66 observations were deleted due to missing data of covariates (**Supplementary Table S12** *)*.

### Impact of donor hypertension on pancreas survival

**Figure 3:**
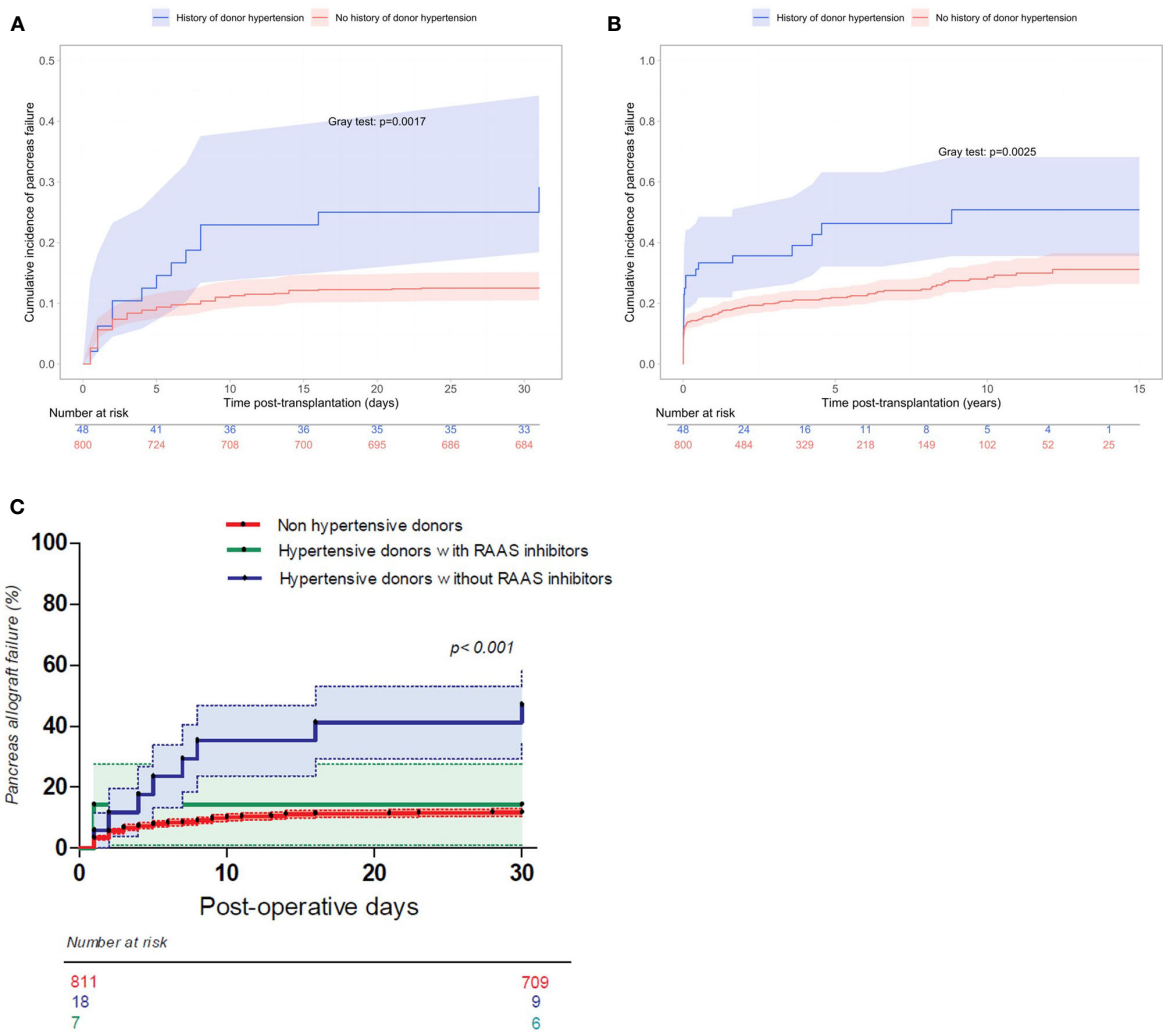
**(A)** Cumulative incidence of pancreas failure within the first 30 days after transplantation according to history of donor hypertension (Aalen-Johansen estimator). **(B)** Cumulative incidence of long-term pancreas failure according to history of donor hypertension (Aalen-Johansen estimator). **(C)** Cumulative incidence of pancreas failure within the first 30 days after transplantation according to history of donor hypertension and RAAS inhibition (Log-rank test).

Hypertension was present in 5.7% of donors (48 out of 848 donors, data were missing for 51 patients). The cumulative incidence of early pancreas failure according to history of hypertension in the donor is presented in **Figure 3A**. Cumulative incidence rate of pancreas failure on postoperative day 30 was 12.6% (95% CI from 10.5% to 15.1%) for donors without hypertension and 26.9% (95% CI from 16.9% to 41.2%) for donors with hypertension.

The cumulative incidence of long-term pancreas failure according to donor hypertension is presented in **Figure 3B**. Cumulative incidence rate of pancreas failure at 10 years was 28.8% (95% CI from 24.7% to 33.3%) %) for donors without hypertension and 50.7% (95% CI from 35.6% to 67.9%)%) for hypertensive donors.

Fifty-six percent of hypertensive donors were receiving anti-hypertensive treatment before death, **Table 3**. Assessing pancreas survival classified according to 3 groups (non-hypertensive donor, hypertensive donor with Renin-Angiotensin-Aldosterone System (RAAS) inhibitors, and hypertensive donor without RAAS inhibitors), allograft survival was significantly worse for patients receiving a pancreas from a hypertensive donor without RAAS inhibitors (50% vs 14%, p < 0.001), **Figure 3C**. On the contrary, occurrence of thrombosis among those who received a pancreas from a hypertensive donor with RAAS inhibitors was similar to that of non-hypertensive donors.

**Table 3:** Descriptive table of patients transplanted from a donor with hypertension (p-values are obtained using Chi-square test or Fisher exact test for categorical variables and using Student’s t-test or Mann-Whitney U for continuous variables).

	All pancreas transplants from HTA donors (n=48)			Allograft failure during the first month (n=14)			Allograft survival at day 30 (n=34)			
	NA	n	%	NA	n	%	NA	n	%	p-value
Treatment using RAAS blocker	23	7	28.0	4	1	10.0	19	6	40.0	0.1718
Hypertensive cardiopathy	16	16	28.1	3	3	27.3	13	6	28.5	1.0000
	NA	Mean	sd	NA	Mean	SD	NA	Mean	SD	p-value
Duration of hypertension (years)	32	3.3	3.0	8	3.2	3.4	24	3.1	2.9	0.9561
Mean arterial pressure at allocation	4	95.2	20.9	0	94.7	21.4	4	95.5	20.8	0.9136

## Discussion

Despite numerous improvements in the selection of the organ donor and the candidate pancreas transplantation, the rate of allograft loss within days after surgery still remain around 10%. According to the French multicenter DIVAT database, we observed among almost 900 pancreas transplant recipients that failures occurring within the first month due to complete thrombosis were 2.5 increased higher for patients receiving a pancreas from a donor with hypertension.

Few studies have evaluated the impact of donor hypertension in pancreas transplantation. A registry analysis suggested poorer long-term outcome if donors were hypertensive (26), but more recently, a large single center cohort did not demonstrate a deleterious effect of donor hypertension in the pancreas (27). However, the endpoint regarding pancreas failure differed, and thrombosis represented only 35% of failures considered during the first 3 months which might explain this discrepancy.

In our study, donor hypertension also influenced long-term pancreas outcome, but this association was restricted to patients with early failure within the first 30 postoperative days. This observation suggests that other mechanisms than those usually described in hypertension-induced arteriosclerosis are involved, linking angiotensin II and thrombosis, especially in this young donor population. The lack of influence of donor hypertension on kidney allograft survival among the SPK group supports this hypothesis, as for the reversal effect observed in hypertensive donors treated with RAAS inhibitors.

The relationship between hypertension and diabetes (i.e. endocrine pancreatic dysfunction) has been well studied. If the risk of developing diabetes in hypertensive patients is higher (28), several studies have focused on the intrinsic role of hypertension into the pancreas. The activation of the RAAS can induce beta-cell injury, in part related to vasoconstriction (29), but mostly via inflammatory pathways involving IL-6 and IL-1β (30, 31). In islet transplantation beta-cell injury has been linked to a release of Tissue Factor, which can lead to thrombotic complications. Moreover, expression of a pro-thrombotic phenotype in endothelial cells due to angiotensin-II have been extensively studied (32–34). However, the transposition in the context of pancreas transplantation remains to be demonstrated. Interestingly, in our cohort, pancreas survival from hypertensive donors treated with RAAS blockers was comparable to that of non-hypertensive donors, supporting the angiotensin II driven hypothesis. However, we acknowledge that the small number of studied cases and missing data regarding donor’s hypertensive therapy does not allow a definitive conclusion to be drawn and further confirmation is required.

As detailed in the literature, donor obesity is also associated with an increased risk of early failure (5). Multiple factors can support this correlation such as pancreatic steatosis, higher intra-abdominal pressure (35) as well as graft injuries induced by an increased secretion of pro-inflammatory cytokines (36). Moreover, obesity is well described risk factor for thrombotic complications through several mechanisms (37).

Thus, our results open the debate on underlying possible new mechanisms of acute thrombosis following pancreas transplantation. If microvascular abnormalities and hemodynamic parameters are undoubtedly involved in this complication due to the particular vasculature of the pancreas (38), other inflammatory processes are possibly associated (39). A better understanding of these pathological or adaptive mechanisms may lead to improve organ preservation (40) and immunomodulation (41, 42) which may facilitate a decrease in early allograft failure.

We believe that our findings may assist transplant physicians and surgeons during organ allocation especially if other well-known risk factors of early allograft failure are present. We believe that hypertension in the donor should not be a reason in itself to discard a pancreatic organ for transplantation, but that it should lead physicians to adapt their post-operative strategy regarding prevention and screening of thrombosis. One approach might be to administer peri-operative anticoagulation and even consider additional anti-inflammatory drug, even if such strategies would require prospective clinical validation.

Our observational study suffers from limitations. First, one cannot exclude possible unobserved confounders. Secondly, we did not include patients with missing data on the covariates, and this has probably reduced statistical power due to a lower sample size. For the analysis of the main outcome, it concerns 113 patients, i.e. less than 15% of the whole sample. However, there is no reason to believe that a selection bias has been introduced since the included and excluded patients were relatively comparable.

In conclusion, pancreas transplantation from donors with history of hypertension was associated with an independent increased risk of early allograft thrombosis and failure within the first month after surgery. The activation of RAAS seems to play a major role in this pathogenic condition, as the risk of thrombosis was reduced in hypertensive donors treated with RAAS inhibitors. The search for physiological and/or endothelial specific characteristics in pancreas from hypertensive donors may allow to further propose allograft pre-treatment in order to avoid this dramatic complication.

## Data availability statement

The data analyzed in this study is subject to the following licenses/restrictions: Data are available upon reasonable request to the corresponding author. Requests to access these datasets should be directed to christophe.masset@chu-nantes.fr.

## Ethics statement

The studies involving humans were approved by Commission nationale de l’informatique et des libertés n°914184. The studies were conducted in accordance with the local legislation and institutional requirements. The participants provided their written informed consent to participate in this study.

## Author contributions

CM: Conceptualization, Formal analysis, Investigation, Writing – original draft, Writing – review & editing. JB: Writing – review & editing. FB: Writing – review & editing. GK: Writing – review & editing. MR: Formal analysis, Writing – review & editing. KR: Formal analysis, Writing – review & editing. FLB: Data curation, Formal analysis, Methodology, Writing – review & editing. LB: Writing – review & editing. XM: Writing – review & editing. CL: Writing – review & editing. DG: Writing – review & editing. CA: Writing – review & editing. MG: Writing – review & editing. JD: Supervision, Writing – review & editing. DC: Supervision, Writing – review & editing.

## Group member of DIVAT Consortium

Lyon E. Hériot: Lionel Badet, Maria Brunet, Fanny Buron, Rémi Cahen, Ricardo Codas, Sameh Daoud, Valérie Dubois, Coralie Fournie, Arnaud Grégoire, Alice Koenig, Charlène Lévi, Emmanuel Morelon, Claire Pouteil-Noble, Maud Rabeyrin, Thomas Rimmelé, Olivier Thaunat; Nantes: Gilles Blancho, Julien Branchereau, Diego Cantarovich, Agnès Chapelet, Jacques Dantal, Clément Deltombe, Lucile Figueres, Raphael Gaisne, Claire Garandeau, Magali Giral, Caroline Gourraud-Vercel, Maryvonne Hourmant, Georges Karam, Clarisse Kerleau, Delphine Kervella, Christophe Masset, Aurélie Meurette, Simon Ville, Christine Kandell, Anne Moreau, Karine Renaudin, Florent Delbos, Alexandre Walencik, Anne Devis; Paris-Necker: Lucile Amrouche, Dany Anglicheau, Olivier Aubert, Lynda Bererhi, Christophe Legendre, Alexandre Loupy, Frank Martinez, Arnaud Méjean, Rébecca Sberro-Soussan, Anne Scemla, Marc-Olivier Timsit, Julien Zuber; Paris Saint Louis: Gillian Divard, Carmen Lefaucheur, Denis Glotz.
