# Supplementary material for: The role of donor hypertension and angiotensin II in the occurrence of early pancreas allograft thrombosis
Source: Front Immunol. 2024 May 17;15:1359381. doi: 10.3389/fimmu.2024.1359381 (PMC11170105; doi:10.3389/fimmu.2024.1359381)
Supplement: Supplementary file 1 [file Table_1.docx]

**Supplementary data**

**Table S1.** Descriptive table of studied patients versus those excluded because of missing data in the pancreas graft failure analysis (p-values are obtained using Chi-square test or Fisher exact test (‡) for categorical variables and using Student’s t-test or Mann-Whitney U (¥) for continuous variables).

|  | **Whole cohort (n=899)** | | | **Studied patients (n=786)** | | | **Excluded patients for missing data (n=113)** | | | **p-value** |
| --- | --- | --- | --- | --- | --- | --- | --- | --- | --- | --- |
|  | **n** | **n** | **%** | **NA** | **n** | **%** | **NA** | **n** | **%** |  |
| **Type of graft** | 0 |  |  | 0 |  |  | 0 |  |  | 0.1454 |
| *SPK* |  | 784 | 87.2 |  | 690 | 87.8 |  | 97 | 85.8 |  |
| *PAK* |  | 55 | 6.1 |  | 43 | 5.5 |  | 11 | 9.7 |  |
| *PTA* |  | 60 | 6.7 |  | 53 | 6.7 |  | 5 | 4.4 |  |
| **Male recipient** | 0 | 510 | 56.7 | 0 | 445 | 56.6 | 0 | 68 | 60.2 | 0.4745 |
| **Re-transplantation** | 0 | 59 | 6.6 | 0 | 46 | 5.9 | 0 | 11 | 9.7 | 0.1133 |
| **History of hypertension** | 0 | 762 | 84.7 | 0 | 677 | 86.1 | 0 | 84 | 74.3 | 0.0011 |
| **History of vascular disease** | 0 | 211 | 23.5 | 0 | 187 | 23.8 | 0 | 25 | 22.1 | 0.6962 |
| **History of cardiac disease** | 0 | 196 | 21.8 | 0 | 171 | 21.8 | 0 | 25 | 22.1 | 0.9294 |
| **Pancreas conservation fluid preservation fluid** | 44 |  |  | 26 |  |  | 19 |  |  | <0.0001 |
| *Celsior* |  | 237 | 27.8 |  | 221 | 29.1 |  | 16 | 17.0 |  |
| *IGL* |  | 320 | 37.4 |  | 302 | 39.7 |  | 20 | 21.3 |  |
| *Other* |  | 298 | 34.8 |  | 237 | 31.2 |  | 58 | 61.7 |  |
| **Male donor** | 2 | 586 | 65.3 | 0 | 508 | 64.6 | 2 | 75 | 67.6 | 0.5437 |
| **Exocrine derivation** | 326 |  |  | 243 |  |  | 158 |  |  | 0.7145 |
| *Enteric with Roux* |  | 269 | 47.0 |  | 261 | 54.4 |  | 9 | 50.0 |  |
| *Enteric without Roux* |  | 226 | 39.4 |  | 219 | 45.6 |  | 9 | 50.0 |  |
| **Vascular cause of donor death** | 3 | 341 | 38.0 | 0 | 297 | 37.8 | 3 | 45 | 40.9 | 0.5277 |
| **Donor hypertension history** | 51 | 48 | 5.7 | 0 | 45 | 5.7 | 51 | 3 | 4.8 | 1.0000‡ |
| **History of donor dyslipidemia** | 237 | 13 | 1.9 | 154 | 12 | 1.9 | 83 | 1 | 3.3 | 0.4559‡ |
| **History of cardiac arrest before sampling** | 8 | 150 | 16.8 | 2 | 142 | 18.1 | 6 | 7 | 6.5 | 0.0026 |
| **Vasopressive drug** | 40 | 753 | 87.6 | 20 | 666 | 86.9 | 19 | 88 | 93.6 | 0.0633 |
| **HLA-A-B-DR incompatibilities > 4** | 7 | 430 | 48.2 | 3 | 629 | 80.3 | 4 | 87 | 79.8 | 0.8992 |
| **Depleting induction** | 7 | 848 | 95.1 | 3 | 757 | 96.7 | 4 | 91 | 83.5 | <0.0001 |
|  | **NA** | **m** | **SD** | **NA** | **m** | **SD** | **NA** | **m** | **SD** | **p-value** |
| **Recipient age (years)** | 1 | 39.7 | 7.9 | 0 | 39.8 | 7.9 | 0 | 38.6 | 8.1 | 0.1228 |
| **Recipient BMI (kg/m²)** | 10 | 22.8 | 3.1 | 3 | 22.8 | 3.2 | 7 | 22.6 | 2.9 | 0.4466 |
| **Pancreas cold ischemia time (min)** | 83 | 674 | 163 | 0 | 11.2 | 2.7 | 82 | 11.3 | 2.7 | 0.8830 |
| **Kidney ischemia time in SPK (min)** | 78 | 790 | 179 | 62 | 13.3 | 3.0 | 12 | 12.4 | 2.9 | 0.0031 |
| **Duration in post-op ICU (days)** | 280 | 2.5 | 3.7 | 192 | 2.5 | 3.7 | 86 | 2.1 | 3.1 | 0.0446¥ |
| **Donor age (years)** | 4 | 32.5 | 10.4 | 0 | 32.5 | 10.4 | 4 | 32.8 | 10.0 | 0.8026 |
| **Donor BMI (kg/m²)** | 34 | 22.9 | 2.9 | 0 | 22.9 | 2.9 | 34 | 22.8 | 3.0 | 0.8842 |
| **Donor eGFR (MDRD, ml/min/m^2^)** | 27 | 111 | 44 | 5 | 119 | 13.7 | 22 | 118 | 12.4 | 0.5559 |
| **Donor lipasemia (IU/L)** | 286 | 63 | 393 | 207 | 58.9 | 385.2 | 78 | 139.2 | 511.8 | 0.3676 |
| BMI, body mass index; CMV, cytomegalovirus; DSA, donor-specific antibodies; eGFR, estimated glomerular filtration rate; HLA, human leucocyte antigens; ICU, intensive care unit; NA: not available (missing); PAK, pancreas after kidney; PTA, pancreas transplant alone; SD, standard deviation; SPK, simultaneous pancreas-kidney. | | | | | | | | | | |

**Table S2.** Univariate Cox model associated with the risk of pancreas graft failure within the first 30 days post-transplantation (n= 899, with 121 events observed during the follow-up).

|  | **NA** | **HR** | **95% CI** | **p-value** |
| --- | --- | --- | --- | --- |
| **Type of graft** | 0 |  |  | 0.5230 |
| *PAK (vs. SPK)* |  | 1.50 | [0.74 ; 3.01] |  |
| *PTA (vs. SPK)* |  | 1.11 | [0.51 ; 2.42] |  |
| **Male recipient** | 0 | 1.08 | [0.75 ; 1.55] | 0.6827 |
| **Recipient age (years)** | 0 | 1.02 | [0.99 ; 1.04] | 0.1527 |
| **Recipient BMI (kg/m²)** | 10 | 1.05 | [0.99 ; 1.11] | 0.1323 |
| **Re-transplantation** | 0 | 1.52 | [0.79 ; 2.93] | 0.2104 |
| **History of hypertension** | 0 | 1.23 | [0.73 ; 2.09] | 0.4412 |
| **History of vascular disease** | 0 | 1.21 | [0.79 ; 1.83] | 0.3806 |
| **History of cardiac disease** | 0 | 0.92 | [0.59 ; 1.44] | 0.7109 |
| **Pancreas cold ischemia time (hours)** | 197 | 1.09 | [1.00 ; 1.17] | 0.0390 |
| **Pancreas preservation** | 45 |  |  | 0.3461 |
| *IGL (vs. Celsior)* |  | 0.78 | [0.44 ; 1.37] |  |
| *Other (vs. Celsior)* |  | 0.68 | [0.41 ; 1.14] |  |
| **Donor age (years)** | 4 | 1.02 | [1.00 ; 1.04] | 0.0661 |
| **Male donor** | 2 | 1.05 | [0.72 ; 1.53] | 0.7946 |
| **Vascular cause of donor death** | 3 | 1.08 | [0.74 ; 1.56] | 0.6927 |
| **Donor BMI (kg/m²)** | 34 | 1.13 | [1.06 ; 1.20] | 0.0001 |
| **Donor history of hypertension** | 45 | 2.40 | [1.36 ; 4.23] | 0.0025 |
| **Donor history of dyslipidemia** | 237 | 0.68 | [0.09 ; 4.96] | 0.7078 |
| **History of cardiac arrest** | 8 | 0.75 | [0.44 ; 1.28] | 0.2953 |
| **Donor eGFR calculated using the CKD-EPI formula** | 27 | 1.00 | [0.99 ; 1.01] | 0.9428 |
| **Vasopressive drug** | 39 | 0.85 | [0.49 ; 1.47] | 0.5511 |
| **Donor lipasemia (IU/L)** | 285 | 1.00 | [1.00 ; 1.00] | 0.7861 |
| **HLA-A-B-DR incompatibilities > 4** | 7 |  |  | 0.7687 |
| **Depleting induction at transplantation** | 7 | 0.94 | [0.41 ; 2.15] | 0.8891 |
| BMI, body mass index; CI, confidence interval; CMV, cytomegalovirus; DSA, donor-specific antibodies; eGFR, estimated glomerular filtration rate; HLA, human leucocyte antigens; HR, hazard ratio; NA: not available (missing); PAK, pancreas after kidney; PTA, pancreas transplant alone; SPK, simultaneous pancreas-kidney. The models were stratified on the center. | | | | |

**Table S3.** Univariate Cox model associated with the risk of long-term pancreas graft failure (n= 899, with 219 events observed during the follow-up).

|  | **NA** | **HR** | **95% CI** | **p-value** |
| --- | --- | --- | --- | --- |
| **Type of graft** | 0 |  |  | 0.0016 |
| *PAK (vs. SPK)* |  | 1.61 | [0.98 ; 2.64] |  |
| *PTA (vs. SPK)* |  | 2.11 | [1.36 ; 3.29] |  |
| **Male recipient** | 0 | 1.04 | [0.80 ; 1.37] | 0.7541 |
| **Recipient age (years)** | 0 | 1.00 | [0.99 ; 1.02] | 0.5719 |
| **Recipient BMI (kg/m²)** | 10 | 1.04 | [1.00 ; 1.09] | 0.0464 |
| **Re-transplantation** | 0 | 1.59 | [1.00 ; 2.54] | 0.0506 |
| **History of hypertension** | 0 | 1.15 | [0.77 ; 1.70] | 0.4984 |
| **History of vascular disease** | 0 | 1.16 | [0.85 ; 1.57] | 0.3424 |
| **History of cardiac disease** | 0 | 0.85 | [0.61 ; 1.19] | 0.3563 |
| **Pancreas cold ischemia time (hours)** | 82 | 1.02 | [0.97 ; 1.08] | 0.3963 |
| **Pancreas preservation** | 45 |  |  | 0.3339 |
| *IGL (vs. Celsior)* |  | 0.84 | [0.55 ; 1.29] |  |
| *Other (vs. Celsior)* |  | 0.77 | [0.54 ; 1.09] |  |
| **Donor age (years)** | 4 | 1.01 | [1.00 ; 1.03] | 0.0277 |
| **Male donor** | 2 | 1.08 | [0.82 ; 1.43] | 0.5813 |
| **Vascular cause of donor death** | 3 | 1.14 | [0.87 ; 1.49] | 0.3488 |
| **Donor BMI (kg/m²)** | 34 | 1.08 | [1.03 ; 1.13] | 0.0017 |
| **Donor history of hypertension** | 51 | 2.15 | [1.35 ; 3.43] | 0.0013 |
| **Donor history of dyslipidemia** | 237 | 1.40 | [0.51 ; 3.80] | 0.5117 |
| **History of cardiac arrest** | 8 | 0.70 | [0.46 ; 1.05] | 0.0847 |
| **eGFR calculated using the CKD-EPI formula** | 27 | 1.00 | [1.00 ; 1.01] | 0.3869 |
| **Vasopressive drug** | 39 | 0.70 | [0.48 ; 1.03] | 0.0675 |
| **Donor lipasemia (IU/L)** | 285 | 1.00 | [1.00 ; 1.00] | 0.7122 |
| **HLA-A-B-DR incompatibilities > 4** | 7 | 0.91 | [0.66 ; 1.26] | 0.5713 |
| **Depleting induction at transplantation** | 7 | 0.78 | [0.42 ; 1.46] | 0.4388 |
| BMI, body mass index; CI, confidence interval; CMV, cytomegalovirus; DSA, donor-specific antibodies; eGFR, estimated glomerular filtration rate; HLA, human leucocyte antigens; HR, hazard ratio; NA: not available (missing); PAK, pancreas after kidney; PTA, pancreas transplant alone; SPK, simultaneous pancreas-kidney. The models were stratified on the center. | | | | |

**Table S4.** Multivariable cause-specific Cox model associated with the risk of long-term pancreas graft failure (n= 786, 113 observations removed because of missing data).

|  | **HR** | **95% CI** | **p-value** |
| --- | --- | --- | --- |
| **Type of graft** |  |  | 0.0003 |
| *PAK (vs. SPK)* | 1.57 | [0.88 ; 2.81] |  |
| *PTA (vs. SPK)* | 2.52 | [1.59 ; 3.98] |  |
| **Pancreas cold ischemia time (hours)** | 1.02 | [0.96 ; 1.08] | 0.4803 |
| **Donor age (years)** | 1.01 | [0.99 ; 1.02] | 0.2524 |
| **Donor BMI (kg/m²)** | 1.07 | [1.01 ; 1.12] | 0.0131 |
| **Donor history of hypertension** | 1.88 | [1.13 ; 3.12] | 0.0143 |
| BMI, body mass index; CI, confidence interval. The model was stratified on the center. | | | |

**Table S5.** Results of the univariate Cox models associated with the risk of pancreas graft failure after Day 30 post-transplantation (n=760, with 98 events observed during the follow-up).

|  | **NA** | **HR** | **95% CI** | **p-value** |
| --- | --- | --- | --- | --- |
| **Type of graft** | 38 |  |  | 0.0001 |
| SPK preemptive (vs. SPK after dialysis) |  | 1.51 | [0.76 ; 3.01] |  |
| PAK (vs. SPK after dialysis) |  | 1.96 | [0.95 ; 4.06] |  |
| PTA (vs. SPK after dialysis) |  | 3.83 | [2.16 ; 6.80] |  |
| **Male recipient** | 0 | 1.00 | [0.67 ; 1.50] | 0.9910 |
| **Recipient age (years)** | 0 | 0.99 | [0.96 ; 1.02] | 0.4409 |
| **Recipient BMI (kg/m²)** | 7 | 1.04 | [0.98 ; 1.11] | 0.1918 |
| **Re-transplantation** | 0 | 1.67 | [0.86 ; 3.25] | 0.1291 |
| **History of hypertension** | 0 | 1.04 | [0.58 ; 1.88] | 0.8922 |
| **History of vascular disease** | 0 | 1.11 | [0.71 ; 1.73] | 0.6478 |
| **History of cardiac disease** | 0 | 0.78 | [0.47 ; 1.30] | 0.3391 |
| **Pancreas ischemia time (hours)** | 63 | 0.98 | [0.91 ; 1.06] | 0.6426 |
| **Pancreas conservation fluid** | 35 |  |  | 0.7981 |
| *IGL (vs. Celsior)* |  | 0.90 | [0.46 ; 1.75] |  |
| *Other (vs. Celsior)* |  | 0.85 | [0.52 ; 1.37] |  |
| **Donor age (years)** | 3 | 1.01 | [0.99 ; 1.03] | 0.2074 |
| **Male donor** | 2 | 1.12 | [0.74 ; 1.70] | 0.5939 |
| **Vascular cause of donor death** | 2 | 1.22 | [0.81 ; 1.82] | 0.3390 |
| **Donor BMI (kg/m²)** | 27 | 1.01 | [0.94 ; 1.09] | 0.6984 |
| **Donor hypertension history** | 43 | 1.57 | [0.68 ; 3.61] | 0.2912 |
| **History of donor dyslipidemia** | 202 | 2.14 | [0.67 ; 6.88] | 0.2016 |
| **History of cardiac arrest before sampling** | 6 | 0.62 | [0.32 ; 1.20] | 0.1552 |
| **eGFR calculated using the CKD-EPI formula** | 23 | 1.01 | [1.00 ; 1.02] | 0.2320 |
| **Vasopressive drug** | 32 | 0.58 | [0.34 ; 0.98] | 0.0427 |
| **Donor lipasemia (IU/L)** | 254 | 1.00 | [1.00 ; 1.00] | 0.8010 |
| **HLA-A-B-DR incompatibilities > 4** | 3 | 0.99 | [0.60 ; 1.62] | 0.9637 |
| **Depleting induction at transplantation** | 4 | 0.58 | [0.23 ; 1.50] | 0.2602 |
| BMI, body mass index; CI, confidence interval; CMV, cytomegalovirus; DSA, donor-specific antibodies; eGFR, estimated glomerular filtration rate; HLA, human leucocyte antigens; HR, hazard ratio; NA: not available (missing); PAK, pancreas after kidney; PTA, pancreas transplant alone; SPK, simultaneous pancreas-kidney. The models were stratified on the center. | | | | |

**Table S6.** Results of the multivariable cause-specific Cox model associated with the risk of pancreas graft failure after Day 30 post-transplantation (n=657, 103 observations removed because of missing data).

|  | **HR** | **95% CI** | **p-value** |
| --- | --- | --- | --- |
| **Type of graft** |  |  | <0.0001 |
| *PAK (vs. SPK)* | 1.65 | [0.64 ; 4.23] |  |
| *PTA (vs. SPK)* | 5.03 | [2.76 ; 9.16] |  |
| **Pancreas ischemia time (hours)** | 1.00 | [0.91 ; 1.09] | 0.9456 |
| **Donor age (years)** | 1.01 | [0.99 ; 1.03] | 0.3079 |
| **Donor BMI (kg/m²)** | 0.99 | [0.92 ; 1.07] | 0.8278 |
| **Donor hypertension history** | 1.22 | [0.47 ; 3.15] | 0.6846 |
| BMI, body mass index; CI, confidence interval. The model was stratified on the center. | | | |

**Table S7.** Results of the univariate Cox models associated with the risk of kidney graft failure at long-term (n=787, with 107 events observed during the follow-up).

|  | **NA** | **HR** | **95% CI** | **p-value** |
| --- | --- | --- | --- | --- |
| **Male recipient** | 0 | 0.85 | [0.58 ; 1.25] | 0.4093 |
| **Recipient age (years)** | 0 | 0.99 | [0.96 ; 1.01] | 0.3956 |
| **Recipient BMI (kg/m²)** | 10 | 1.04 | [0.97 ; 1.11] | 0.2821 |
| **Re-transplantation** | 0 | 2.00 | [0.27 ; 14.74] | 0.4974 |
| **History of hypertension** | 0 | 2.18 | [0.94 ; 5.02] | 0.0677 |
| **History of vascular disease** | 0 | 1.24 | [0.81 ; 1.91] | 0.3150 |
| **History of cardiac disease** | 0 | 0.88 | [0.54 ; 1.44] | 0.6097 |
| **Pancreas ischemia time (hours)** | 82 | 0.96 | [0.89 ; 1.03] | 0.2616 |
| **Kidney ischemia time (hours)** | 14 | 0.98 | [0.91 ; 1.05] | 0.4965 |
| **Pancreas conservation fluid** | 45 |  |  | 0.2960 |
| *IGL (vs. Celsior)* |  | 1.70 | [0.85 ; 3.39] |  |
| *Other (vs. Celsior)* |  | 1.31 | [0.79 ; 2.16] |  |
| **Donor age (years)** | 4 | 1.03 | [1.01 ; 1.05] | 0.0107 |
| **Male donor** | 2 | 1.11 | [0.74 ; 1.65] | 0.6225 |
| **Vascular cause of donor death** | 2 | 1.22 | [0.83 ; 1.80] | 0.3133 |
| **Donor BMI (kg/m²)** | 34 | 1.12 | [1.05 ; 1.20] | 0.0007 |
| **Donor hypertension history** | 35 | 1.92 | [1.01 ; 3.66] | 0.0465 |
| **History of donor dyslipidemia** | 214 | 2.37 | [0.57 ; 9.85] | 0.2355 |
| **History of cardiac arrest before sampling** | 5 | 0.96 | [0.53 ; 1.72] | 0.8880 |
| **eGFR calculated using the CKD-EPI formula** | 27 | 0.99 | [0.97 ; 1.01] | 0.3136 |
| **Vasopressive drug** | 21 | 0.55 | [0.33 ; 0.91] | 0.0194 |
| **Donor lipasemia (IU/L)** | 272 | 1.00 | [1.00 ; 1.00] | 0.5213 |
| **HLA-A-B-DR incompatibilities > 4** | 6 | 0.73 | [0.47 ; 1.15] | 0.1759 |
| **Depleting induction at transplantation** | 7 | 0.39 | [0.17 ; 0.92] | 0.0304 |
| BMI, body mass index; CI, confidence interval; CMV, cytomegalovirus; DSA, donor-specific antibodies; eGFR, estimated glomerular filtration rate; HLA, human leucocyte antigens; HR, hazard ratio; NA: not available (missing); PAK, pancreas after kidney; PTA, pancreas transplant alone; SPK, simultaneous pancreas-kidney. The models were stratified on the center. | | | | |

**Table S8.** Results of the multivariable cause-specific Cox model associated with the risk of long-term kidney graft failure (n=737, 50 observations removed because of missing data).

|  | **HR** | **95% CI** | **p-value** |
| --- | --- | --- | --- |
| **Donor age (years)** | 1.02 | [1.00 ; 1.04] | 0.0941 |
| **Donor BMI (kg/m²)** | 1.09 | [1.01 ; 1.17] | 0.0180 |
| **Donor hypertension history** | 1.58 | [0.80 ; 3.10] | 0.1844 |
| BMI, body mass index; CI, confidence interval. The model was stratified on the center. | | | |

**Table S9.** Descriptive table of studied patients versus those excluded because of missing data in the kidney graft failure analysis (p-values are obtained using Chi-square test or Fisher exact test (‡) for categorical variables and using Student’s t-test or Mann-Whitney U (¥) for continuous variables).

|  | **Whole sample (n=787)** | | | **Studied patients (n=737)** | | | **Excluded patients for missing data (n=50)** | | | **p-value** |
| --- | --- | --- | --- | --- | --- | --- | --- | --- | --- | --- |
|  | **NA** | **n** | **%** | **NA** | **n** | **%** | **NA** | **n** | **%** |  |
| **Male recipient** | 0 | 454 | 57.7 | 0 | 423 | 57.4 | 0 | 31 | 62.0 | 0.5236 |
| **Re-transplantation** | 0 | 6 | 0.8 | 0 | 5 | 0.7 | 0 | 1 | 2.0 | 0.3264‡ |
| **History of hypertension** | 0 | 688 | 87.4 | 0 | 643 | 87.2 | 0 | 45 | 90.0 | 0.5698 |
| **History of vascular disease** | 0 | 183 | 23.3 | 0 | 171 | 23.2 | 0 | 12 | 24.0 | 0.8972 |
| **History of cardiac disease** | 0 | 167 | 21.2 | 0 | 155 | 21.0 | 0 | 12 | 24.0 | 0.6193 |
| **Pancreas conservation fluid** | 45 |  |  | 43 |  |  | 2 |  |  | <0.0001 |
| *Celsior* |  | 201 | 27.1 |  | 196 | 28.2 |  | 5 | 10.4 |  |
| *IGL* |  | 303 | 40.8 |  | 295 | 42.5 |  | 8 | 16.7 |  |
| *Other* |  | 238 | 32.1 |  | 203 | 29.3 |  | 35 | 72.9 |  |
| **Male donor** | 2 | 518 | 66.0 | 0 | 486 | 65.9 | 2 | 32 | 66.7 | 0.9183 |
| **Vascular cause of donor death** | 2 | 296 | 37.7 | 0 | 273 | 37.0 | 2 | 23 | 47.9 | 0.1320 |
| **Donor hypertension history** | 35 | 42 | 5.6 | 0 | 40 | 5.4 | 35 | 2 | 13.3 | 0.2022‡ |
| **History of donor dyslipidemia** | 214 | 10 | 1.7 | 174 | 9 | 1.6 | 40 | 1 | 10.0 | 0.1626‡ |
| **History of cardiac arrest before sampling** | 5 | 125 | 16.0 | 1 | 122 | 16.6 | 4 | 3 | 6.5 | 0.0710 |
| **Vasopressive drug** | 21 | 676 | 88.3 | 16 | 633 | 87.8 | 5 | 43 | 95.6 | 0.1168 |
| **HLA-A-B-DR incompatibilities > 4** | 6 | 627 | 80.3 | 5 | 588 | 80.3 | 1 | 39 | 79.6 | 0.9002 |
| **Depleting induction at transplantation** | 7 | 739 | 94.7 | 6 | 703 | 96.2 | 1 | 36 | 73.5 | <0.0001‡ |
|  | **NA** | **m** | **SD** | **NA** | **m** | **SD** | **NA** | **m** | **SD** | **p-value** |
| **Recipient age (years)** | 0 | 39.3 | 7.8 | 0 | 39.4 | 7.8 | 0 | 38.6 | 7.9 | 0.5232 |
| **Recipient BMI (kg/m²)** | 10 | 22.7 | 3.0 | 8 | 22.7 | 3.1 | 2 | 22.6 | 2.6 | 0.8031 |
| **Pancreas ischemia time (hours)** | 82 | 11.2 | 2.7 | 47 | 11.2 | 2.7 | 35 | 10.3 | 1.9 | 0.2141¥ |
| **Kidney ischemia time (hours)** | 14 | 13.2 | 3.0 | 11 | 13.2 | 3.0 | 3 | 12.3 | 3.1 | 0.0510 |
| **Duration in ICU of the post-op recipient (days)** | 264 | 2.6 | 3.8 | 224 | 2.6 | 3.8 | 40 | 2.9 | 3.7 | 0.9494¥ |
| **Donor age (years)** | 4 | 32.9 | 10.1 | 0 | 32.9 | 10.1 | 4 | 32.6 | 9.3 | 0.8495 |
| **Donor BMI (kg/m²)** | 34 | 23.0 | 2.9 | 0 | 23.0 | 2.8 | 34 | 23.2 | 3.5 | 0.6488¥ |
| **eGFR calculated using the CKD-EPI formula** | 27 | 118.3 | 11.2 | 20 | 118.4 | 11.2 | 7 | 117.5 | 11.1 | 0.6321 |
| **Donor lipasemia (IU/L)** | 272 | 66.9 | 428.8 | 230 | 66.8 | 432.1 | 42 | 70.6 | 63.4 | 0.1822¥ |
| BMI, body mass index; CMV, cytomegalovirus; DSA, donor-specific antibodies; eGFR, estimated glomerular filtration rate; HLA, human leucocyte antigens; ICU, intensive care unit; NA: not available (missing); PAK, pancreas after kidney; PTA, pancreas transplant alone; SD, standard deviation; SPK, simultaneous pancreas-kidney. | | | | | | | | | | |

**Table S10.** Results of the univariate Cox models associated with the risk of patient death (n=899, with 85 events observed during the follow-up).

|  | **NA** | **HR** | **95% CI** | **p-value** |
| --- | --- | --- | --- | --- |
| **Type of graft** | 0 |  |  | 0.9708 |
| *PAK (vs. SPK)* |  | 1.01 | [0.40 ; 2.53] |  |
| *PTA (vs. SPK)* |  | 1.12 | [0.45 ; 2.81] |  |
| **Male recipient** | 0 | 1.16 | [0.74 ; 1.81] | 0.5161 |
| **Recipient age (years)** | 0 | 1.05 | [1.02 ; 1.08] | 0.0003 |
| **Recipient BMI (kg/m²)** | 10 | 1.03 | [0.96 ; 1.10] | 0.4206 |
| **Re-transplantation** | 0 | 0.81 | [0.29 ; 2.24] | 0.6861 |
| **History of hypertension** | 0 | 0.88 | [0.47 ; 1.64] | 0.6917 |
| **History of vascular disease** | 0 | 2.04 | [1.31 ; 3.18] | 0.0016 |
| **History of cardiac disease** | 0 | 1.40 | [0.87 ; 2.23] | 0.1619 |
| **Pancreas ischemia time (hours)** | 82 | 0.95 | [0.88 ; 1.03] | 0.2540 |
| **Pancreas conservation fluid** | 45 |  |  | 0.4114 |
| *IGL (vs. Celsior)* |  | 1.67 | [0.78 ; 3.57] |  |
| *Other (vs. Celsior)* |  | 1.17 | [0.70 ; 1.98] |  |
| **Donor age (years)** | 4 | 1.01 | [0.99 ; 1.03] | 0.1907 |
| **Male donor** | 2 | 0.89 | [0.57 ; 1.37] | 0.5953 |
| **Vascular cause of donor death** | 3 | 1.04 | [0.67 ; 1.60] | 0.8749 |
| **Donor BMI (kg/m²)** | 34 | 1.00 | [0.92 ; 1.08] | 0.9492 |
| **Donor hypertension history** | 51 | 1.62 | [0.76 ; 3.43] | 0.2089 |
| **History of donor dyslipidemia** | 237 | 1.06 | [0.15 ; 7.79] | 0.9512 |
| **History of cardiac arrest before sampling** | 8 | 1.50 | [0.86 ; 2.61] | 0.1523 |
| **eGFR calculated using the CKD-EPI formula** | 27 | 0.99 | [0.97 ; 1.01] | 0.2474 |
| **Vasopressive drug** | 39 | 1.07 | [0.53 ; 2.15] | 0.8591 |
| **Donor lipasemia (IU/L)** | 285 | 1.00 | [1.00 ; 1.00] | 0.8874 |
| **HLA-A-B-DR incompatibilities > 4** | 7 | 0.76 | [0.46 ; 1.25] | 0.2711 |
| **Depleting induction at transplantation** | 7 | 0.45 | [0.17 ; 1.18] | 0.1045 |
| BMI, body mass index; CI, confidence interval; CMV, cytomegalovirus; DSA, donor-specific antibodies; eGFR, estimated glomerular filtration rate; HLA, human leucocyte antigens; HR, hazard ratio; NA: not available (missing); PAK, pancreas after kidney; PTA, pancreas transplant alone; SPK, simultaneous pancreas-kidney. The models were stratified on the center. | | | | |

**Table S11.** Results of the multivariable cause-specific Cox model associated with the risk of patient death (n=833, 66 observations removed because of missing data).

|  | **HR** | **95% CI** | **p-value** |
| --- | --- | --- | --- |
| **Recipient age (years)** | 1.05 | [1.02 ; 1.08] | 0.0012 |
| **History of vascular disease** | 2.01 | [1.24 ; 3.26] | 0.0047 |
| **Donor age (years)** | 1.01 | [0.99 ; 1.03] | 0.5050 |
| **Donor BMI (kg/m²)** | 0.98 | [0.90 ; 1.07] | 0.6446 |
| **Donor hypertension history** | 1.56 | [0.72 ; 3.40] | 0.2608 |
| BMI, body mass index; CI, confidence interval. The model was stratified on the center. | | | |

**Table S12.** Descriptive table of studied patients versus those excluded because of missing data in the patient death analysis (p-values are obtained using Chi-square test or Fisher exact test (‡) for categorical variables and using Student’s t-test or Mann-Whitney U (¥) for continuous variables).

|  | **Whole sample (n=899)** | | | **Studied patients (n=833)** | | | **Excluded patients for missing data (n=66)** | | | **p-value** |
| --- | --- | --- | --- | --- | --- | --- | --- | --- | --- | --- |
|  | **NA** | **n** | **%** | **NA** | **n** | **%** | **NA** | **n** | **%** |  |
| **Type of graft** | 0 |  |  | 0 |  |  | 0 |  |  | 0.0019‡ |
| *SPK* |  | 787 | 87.5 |  | 737 | 88.5 |  | 50 | 75.8 |  |
| *PAK* |  | 54 | 6.0 |  | 43 | 5.2 |  | 11 | 16.7 |  |
| *PTA* |  | 58 | 6.5 |  | 53 | 6.4 |  | 5 | 7.6 |  |
| **Male recipient** | 0 | 513 | 57.1 | 0 | 471 | 56.5 | 0 | 42 | 63.6 | 0.2624 |
| **Re-transplantation** | 0 | 57 | 6.3 | 0 | 47 | 5.6 | 0 | 10 | 15.2 | 0.0061‡ |
| **History of hypertension** | 0 | 761 | 84.6 | 0 | 704 | 84.5 | 0 | 57 | 86.4 | 0.6882 |
| **History of vascular disease** | 0 | 212 | 23.6 | 0 | 193 | 23.2 | 0 | 19 | 28.8 | 0.3007 |
| **History of cardiac disease** | 0 | 196 | 21.8 | 0 | 176 | 21.1 | 0 | 20 | 30.3 | 0.0823 |
| **Pancreas conservation fluid** | 45 |  |  | 43 |  |  | 2 |  |  | <0.0001 |
| *Celsior* |  | 237 | 27.8 |  | 228 | 28.9 |  | 9 | 14.1 |  |
| *IGL* |  | 322 | 37.7 |  | 312 | 39.5 |  | 10 | 15.6 |  |
| *Other* |  | 295 | 34.5 |  | 250 | 31.6 |  | 45 | 70.3 |  |
| **Male donor** | 2 | 583 | 65.0 | 0 | 540 | 64.8 | 2 | 43 | 67.2 | 0.7027 |
| **Vascular cause of donor death** | 3 | 342 | 38.2 | 0 | 314 | 37.7 | 3 | 28 | 44.4 | 0.2877 |
| **Donor hypertension history** | 51 | 48 | 5.7 | 0 | 46 | 5.5 | 51 | 2 | 13.3 | 0.2065‡ |
| **History of donor dyslipidemia** | 237 | 13 | 2.0 | 181 | 12 | 1.8 | 56 | 1 | 10.0 | 0.1810‡ |
| **History of cardiac arrest before sampling** | 8 | 149 | 16.7 | 2 | 144 | 17.3 | 6 | 5 | 8.3 | 0.0714 |
| **Vasopressive drug** | 39 | 754 | 87.7 | 22 | 707 | 87.2 | 17 | 47 | 95.9 | 0.0706 |
| **HLA-A-B-DR incompatibilities > 4** | 7 | 716 | 80.3 | 5 | 668 | 80.7 | 2 | 48 | 75.0 | 0.2716 |
| **Depleting induction at transplantation** | 7 | 848 | 95.1 | 6 | 797 | 96.4 | 1 | 51 | 78.5 | <0.0001‡ |
|  | **NA** | **m** | **SD** | **NA** | **m** | **SD** | **NA** | **m** | **SD** | **p-value** |
| **Recipient age (years)** | 0 | 39.7 | 8.0 | 0 | 39.6 | 7.9 | 0 | 40.1 | 8.4 | 0.6740 |
| **Recipient BMI (kg/m²)** | 10 | 22.8 | 3.1 | 8 | 22.8 | 3.1 | 2 | 23.0 | 3.0 | 0.6128 |
| **Pancreas ischemia time (hours)** | 82 | 11.2 | 2.7 | 47 | 11.2 | 2.7 | 35 | 11.3 | 2.7 | 0.8830 |
| **Kidney ischemia time (hours)** | 74 | 13.2 | 3.0 | 68 | 13.2 | 3.0 | 6 | 12.6 | 3.2 | 0.1456 |
| **Duration in ICU of the post-op recipient (days)** | 278 | 2.5 | 3.7 | 233 | 2.5 | 3.7 | 45 | 1.7 | 2.8 | 0.0070¥ |
| **Donor age (years)** | 4 | 32.6 | 10.3 | 0 | 32.5 | 10.4 | 4 | 32.7 | 9.9 | 0.9190 |
| **Donor BMI (kg/m²)** | 34 | 22.9 | 2.9 | 0 | 22.9 | 2.9 | 34 | 22.6 | 3.4 | 0.6749 |
| **eGFR calculated using the CKD-EPI formula** | 27 | 119.1 | 13.6 | 20 | 119.2 | 13.7 | 7 | 117.7 | 12.5 | 0.3934 |
| **Donor lipasemia (IU/L)** | 285 | 63.5 | 393.5 | 236 | 62.8 | 398.8 | 49 | 87.1 | 86.0 | 0.0085¥ |
| BMI, body mass index; CMV, cytomegalovirus; DSA, donor-specific antibodies; eGFR, estimated glomerular filtration rate; HLA, human leucocyte antigens; ICU, intensive care unit; NA: not available (missing); PAK, pancreas after kidney; PTA, pancreas transplant alone; SD, standard deviation; SPK, simultaneous pancreas-kidney. | | | | | | | | | | |
